# Chloral in Trismus

**Published:** 1874-11

**Authors:** 


Hydrate of Chloral in Trismus and Tetanus of New Born Children.—Dr. A. Von Huttenbrenner thinks he is justified in arriving at the following conclusions: 1. Tetanus is a disease which is not necessarily fatal. 2. The cause of the disease is febrile or not; in the former case it is but a partial manifestation of general poisoning of the blood; in the latter it consists of reflex spasms excited by peripheric irritation. 3. Prognosis is more favorable in cases unattended by fever, though it is not necessarily unfavorable in cases accompanied by fever. 4. Chloral hydrate is far from being a specific for tetanus, but its employment must be recommended because it is a pure hypnotic, which does not determine congestion of the brain as morphia does, is easily administered to children, and finally has been undoubtedly successful in a great many cases.—*Fahrb. d. Kinderheilk.*

